# PP1γ2 and PPP1R11 Are Parts of a Multimeric Complex in Developing Testicular Germ Cells in which their Steady State Levels Are Reciprocally Related

**DOI:** 10.1371/journal.pone.0004861

**Published:** 2009-03-20

**Authors:** Lina Cheng, Stephen Pilder, Angus C. Nairn, Shandilya Ramdas, Srinivasan Vijayaraghavan

**Affiliations:** 1 Department of Biological Sciences, Kent State University, Kent, Ohio, United States of America; 2 Department of Anatomy and Cell Biology, Temple University School of Medicine, Philadelphia, Pennsylvania, United States of America; 3 Department of Psychiatry, Yale University, New Haven, Connecticut, United States of America; University of Washington, United States of America

## Abstract

Mice lacking the protein phosphatase 1 gamma isoforms, PP1γ1 and PP1γ2, are male-sterile due to defective germ cell morphogenesis and apoptosis. However, this deficiency causes no obvious abnormality in other tissues. A biochemical approach was employed to learn how expression versus deficiency of PP1γ2, the predominant PP1 isoform in male germ cells, affects spermatogenesis. Methods used in this study include column chromatography, western blot and northern blot analyses, GST pull-down assays, immunoprecipitation, non-denaturing gel electrophoresis, phosphatase enzyme assays, protein sequencing, and immunohistochemistry. We report for the first time that in wild-type testis, PP1γ2 forms an inactive complex with actin, protein phosphatase 1 regulatory subunit 7 (PPP1R7), and protein phosphatase 1 regulatory subunit 11 (PPP1R11), the latter, a potent PP1 inhibitor. Interestingly, PPP1R11 protein, but not its mRNA level, falls significantly in PP1γ-null testis where mature sperm are virtually absent. Conversely, both mature sperm numbers and the PPP1R11 level increase substantially in PP1γ-null testis expressing transgenic PP1γ2. PPP1R11 also appears to be ubiquitinated in PP1γ-null testis. The levels of PP1γ2 and PPP1R11 were increased in phenotypically normal PP1α-null testis. However, in PP1α-null spleen, where PP1γ2 normally is not expressed, PPP1R11 levels remained unchanged. Our data clearly show a direct reciprocal relationship between the levels of the protein phosphatase isoform PP1γ2 and its regulator PPP1R11, and suggest that complex formation between these polypeptides in testis may prevent proteolysis of PPP1R11 and thus, germ cell apoptosis.

## Introduction

All four isoforms of protein phosphatase 1 (PP1), PP1α, PP1β, PP1γ1 and PP1γ2, are expressed in mammalian testis. Targeted disruption of the *Ppp1cc* gene, resulting in ablation of both PP1γ1 and PP1γ2, leads to aberrant sperm morphogenesis, testicular apoptosis, and subsequent male sterility, despite increased “compensatory” expression of PP1α and PP1β [1]. Because PP1γ2 is the only PP1 isoform to exhibit significant expression in differentiating male germ cells, particularly in spermatids [2], [3], its absence could be at the heart of the PP1γ-null phenotype [1]. Thus, PP1γ2 might have a fundamental, isoform-specific role in mammalian spermiogenesis.

PP1γ2 is also the only PP1 isoform detected in mammalian spermatozoa [2], [3], where inhibition of its activity in caudal epididymal sperm has been linked to the onset of progressive motility, and to a significant increase in vigor in already progressively motile sperm [2], [3]. These findings suggest that PP1γ2 is a vital mediator of sperm function in mammals. However, the reason for this is not yet clear since the genomes of eukaryotic species other than mammals do not contain a PP1 orthologue resembling PP1γ2.

What differentiates PP1γ2 from other PP1 isoforms is its distinctive, almost completely conserved 21-amino acid C-terminal extension. Even so, the function of this unique C-terminus remains unknown, as it does not appear to be essential for the catalytic activity of PP1γ2 [4], [5].

A number of PP1 interacting proteins in somatic cells have been detected by a variety of approaches, and considerable progress has been made in defining the functions of these proteins and how they regulate PP1 in those cells [6]–[9]. In comparison, our understanding of the regulation of PP1γ2 in male germ cells is limited primarily to what we have learned about generic PP1 function in somatic cells. Yeast two-hybrid approaches have identified several PP1 interacting proteins in testis [10]–[13]. However, the dimunition/termination of transcriptional and translational activities in haploid spermatids and terminally differentiated testicular spermatozoa make the application of this technique ineffectual in determining the role of PP1γ2 and its binding partners in these cell types. Thus, to elucidate the functional interactions of PP1γ2 with its regulators and other substrates in developing and mature male gametes, biochemical approaches are generally employed [1]–[3], [14]–[20].

To date, such studies have shown that sperm do not appear to contain any detectable PPP1R1 (phosphoprotein phosphatase 1 regulatory subunit 1, inhibitor 1, I1), a PP1 regulatory subunit whose activity is controlled by protein kinase A phosphorylation [21], [22]. Sperm do contain inhibitor activity similar to that of PPP1R2 (phosphoprotein phosphatase 1 regulatory subunit 2, inhibitor 2, I2), mediated by GSK3 (glycogen synthase kinase 3) phosphorylation [1], [2]. Experiments, using column/affinity purification techniques with anti-PP1γ2 antibodies have demonstrated that sperm contain the homologue of the yeast PP1 binding protein, PPP1R7 (phosphoprotein phosphatase 1 regulatory subunit 7, Sds22) [14]. The protein Sds22 was originally identified in yeast as a positive regulator of protein phosphatase-1, required for the mitotic metaphase/anaphase transition [23]–[25]. Nonetheless, in cultured mammalian cells, a partial inhibitory effect on a recombinant catalytic subunit of PP1 by a synthetic polypeptide corresponding to the Sds22 sixth leucine-rich repeat (LRR) has been demonstrated [26]. Additionally, characterization of the Sds22-PP1γ2 complex in spermatozoa revealed that the complex was catalytically inactive [14]. Thus, Sds22 is currently believed to be an inhibitor of PP1 in mammals. However, other components in the male germ cell complex containing Sds22 and PP1γ2 have not yet been identified.

Finally, a potent heat-stable PP1 inhibitor, PPP1R11 (phosphoprotein phosphatase 1 regulatory subunit 11, inhibitor 3, I3), first discovered in yeast, and later in human brain through yeast two-hybrid studies [27], was identified subsequently as the orthologue of the mouse *t* complex testis-expressed gene, *Tctex5* (*t* complex testis expressed gene 5). Its PP1 inhibitory power has since been demonstrated against both recombinant Mn^2+^-dependent and metal-independent isoforms of PP1α [28]. A recent study suggests that cleavage of this PP1 inhibitor by Caspase-3 in actinomyosin D-treated HL60 cells promotes apoptosis [9].

Other recent studies in yeast and mammalian cell culture have suggested that Sds22, PPP1R11, and PP1α form a trimeric complex in which PP1α is catalytically inactive [29], [30]. The physiological role of the complex between PP1, Sds22, and PPP1R11, and whether such a complex exists in developing male germ cells are not known. However, the conserved presence of PPP1R11 and Sds22 in a broad range of eukaryotic organisms and tissues [31], including the mammalian testis, suggests that both may be vital regulators of PP1 activity in the male gonad.

In this study, we demonstrate the formation of a complex between PP1γ2, PPP1R11, Sds22, and actin in both wild-type differentiating and terminally differentiated male germ cells, in which PP1γ2 appears to be held in a catalytically inactive state. We also show evidence that PP1γ2 has an anti-apoptotic effect in the testis, and explore the possibility that this result could be mediated by its ability to enhance the stability of PPP1R11.

## Results

### PPP1R11 mRNA and protein were abundantly expressed in testis compared to other tissues

**Figure 1:**
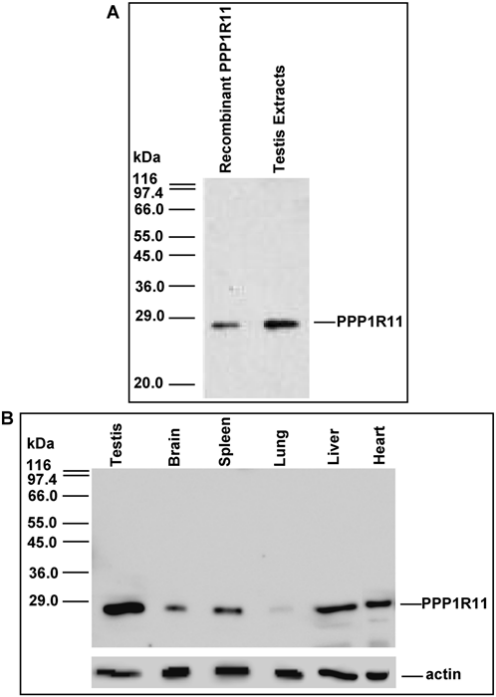
PPP1R11 is present in various tissues including testis. *A*. Validation of PPP1R11 antibody was performed as follows: recombinant PPP1R11 and testis protein extracts were separated by SDS-PAGE followed by western blot analysis with affinity purified rabbit polyclonal anti-PPP1R11. Size markers (left) were derived from β-Galactosidase (116-kDa), Phosphorylase b (97.4-kDa), Albumin (66-kDa), Glutamic dehydrogenase (55-kDa), Ovalbumin (45-kDa), Glyceraldehyde-3-phosphate dehydrogenase (36-kDa), Carbonic anhydrase (29-kDa), and Soybean trypsin inhibitor (20-kDa). *B*. Soluble protein extracts from testis and somatic tissues were separated by SDS-PAGE followed by western blot analysis with the validated affinity purified PPP1R11 antibody, and the same blot was stripped and reprobed with anti-actin used as a loading control.

A previous study documented the presence of *ppp1r11* mRNA in testis, and showed that the steady state level of this message is clearly higher in testis than in other tissues [32]. We confirmed, by northern blot analysis of mRNAs isolated from testis and numerous other tissues, that *ppp1r11* testicular mRNA is significantly more abundant than it is in other tissues (data not shown). To determine if the steady state levels of PPP1R11 protein in various tissues reflect these mRNA results, we prepared an affinity-purified polyclonal antibody (see Materials and Methods), and demonstrated that it specifically recognized both recombinant PPP1R11 and endogenous testicular PPP1R11 (Fig. 1A). Subsequent western blots of protein extracts from testis and various somatic tissues probed with our PPP1R11 antibody, established the existence of a single immunoreactive protein at 27-kDa at significantly higher levels in testis than in other tissues (Fig. 1B).

### PPP1R11 binds to testis-expressed PP1γ2 and Sds22 both in vitro and in vivo

**Figure 2:**
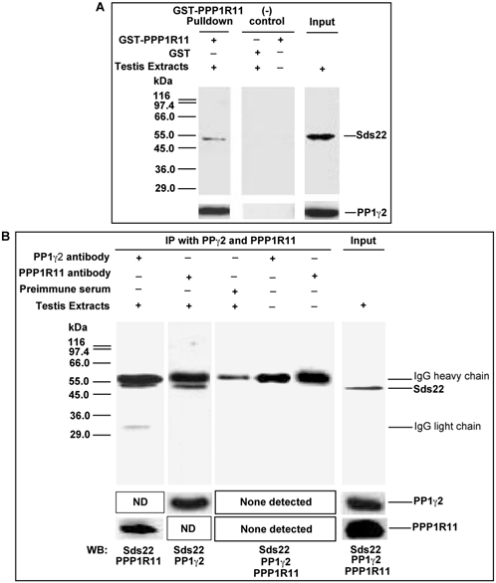
Sds22, PPP1R11, and PP1γ2 are bound to each other in crude testis protein extracts. *A*. The recombinant proteins GST-PPP1R11 and control GST were incubated with testis cell lysates in the presence of Glutathione-Sepharose beads. The eluted proteins and testis extracts alone were resolved by SDS-PAGE and subjected to western blot analysis with anti-Sds22 and anti-PP1γ2 antibodies. *B*. Testis protein extracts and buffer controls were incubated with anti-PP1γ2, anti-PPP1R11, or preimmune serum immobilized on Protein G-Sepharose-4 beads, as indicated at the top of the figure. The immunoprecipitates were separated by SDS-PAGE and immunoblotted for the proteins indicated at the bottom of the figure. (ND: not done).

The protein PPP1R11 has been reported to co-localize with PP1γ1 in nucleoli and with PP1α in centrosomes in somatic cells, and has been co-precipitated with PP1γ1 and PP1α but not PP1β in HEK cells [28]. However, it was not known whether PPP1R11 binds PP1γ2 in testicular germ cells. To determine if PPP1R11 is capable of binding to PP1γ2 *in vitro*, we made GST-tagged recombinant PPP1R11 protein in bacteria, immobilized it on glutathione Sepharose 4B beads, and incubated it with mouse testis extracts. After extensive washing, bound proteins released by reduced glutathione were analyzed on western blots probed with anti-PP1γ2 and anti-Sds22 antibodies. Figure 2A demonstrates that PP1γ2 from testis extracts was bound to GST-PPP1R11 but not to GST alone. Not surprisingly, Sds22 was also identified as a potential PPP1R11 and/or PP1γ2 binding protein in testis extracts (Fig. 2A).

Immunoprecipitation was used to further explore whether endogenous PPP1R11 is bound to PP1γ2 and/or Sds22. Proteins immunoprecipitated by antibodies to PPP1R11 or PP1γ2 were separated by SDS-PAGE, and gels were analyzed by western blotting with anti-PP1γ2 and anti-Sds22 antibodies as probes for proteins precipitated by anti-PPP1R11, and anti-PPP1R11 and anti-Sds22 antibodies as probes for proteins precipitated by anti-PP1γ2. The results established that anti-PPP1R11 co-precipitated both PP1γ2 and Sds22, whereas anti-PP1γ2 co-precipitated both PPP1R11 and Sds22 from mouse testis extracts (Fig. 2B). Taken together with our GST-pull down results, these data strongly suggest that PPP1R11, Sds22, and PP1γ2 are part of a single complex or exist as multiple pairs of heterodimers in testis.

**Figure 3:**
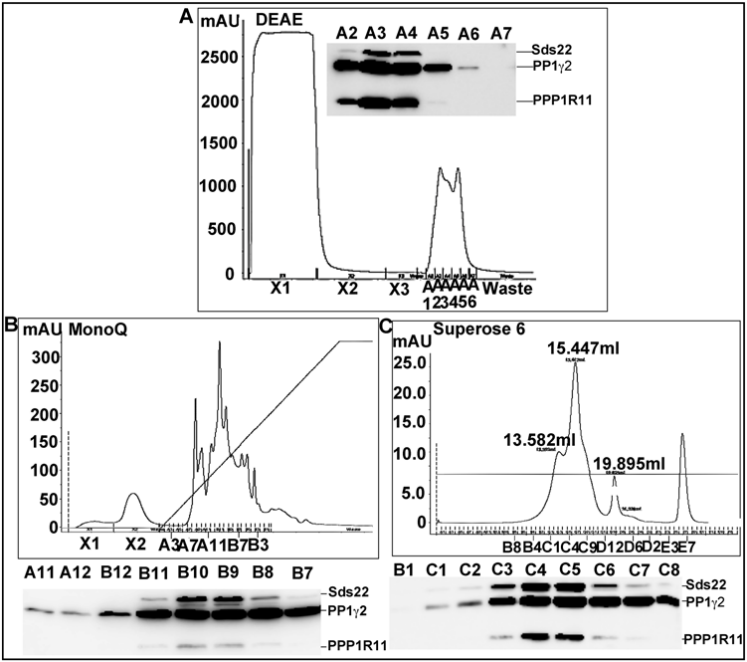
PPP1R11, Sds22 and PP1γ2 co-elute during chromatographic purification of testis extracts. Western blot analysis of purified column fractions shows a consistent co-elution pattern of testicular PP1γ2, Sds22, and PPP1R11 through a series of chromatographic media. *A*. DEAE chromatography showing PP1γ2, Sds22, and PPP1R11 co-eluting primarily in fractions A3 and A4. *B*. A3 and A4 fractions from the DEAE column were pooled and purified by MonoQ column fractionation. PP1γ2, Sds22, and PPP1R11 co-eluted in fractions B8–11. *C*. B8–B11 fractions from the MonoQ column were pooled and purified by Superose 6 column chromatography, from which PP1γ2, Sds22, and PPP1R11 co-eluted from 14.05–15.05 ml. In each instance 0.5 ml of co-eluting fractions were collected and concentrated, then assayed by SDS-PAGE/western blot analysis.

To further clarify these binding relationships *in vivo*, chromatographic fractionation of mouse testis protein extracts was performed to purify the putative complex(es) containing PPP1R11, PP1γ2 and Sds22. Column fractions were analyzed for PPP1R11, PP1γ2, and Sds22 immunoreactivities. The presence and abundance of all three proteins in DEAE, MonoQ, and Superose 6 column fractions is shown in Figure 3A, B, and C, respectively, providing further evidence that the three proteins may be part or all of a single soluble complex *in vivo*.

**Figure 4:**
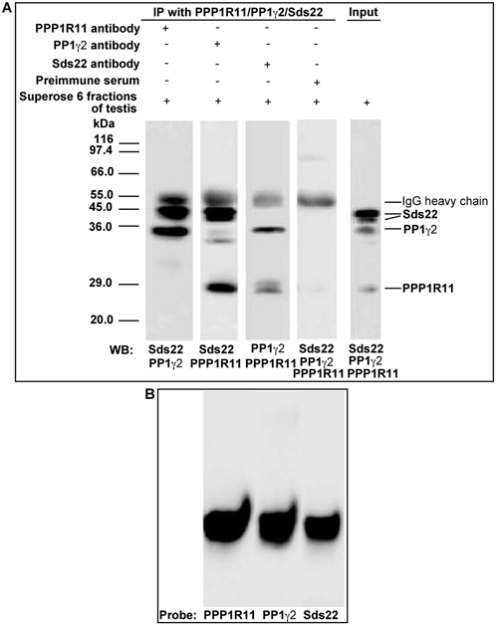
PPP1R11, Sds22, and PP1γ2 from co-eluting fractions are reciprocally co-immunoprecipitated, and co-migrate by native PAGE. *A*. Superose 6 fractions (C4 and C5 from Figure 3C) of testis extracts containing co-eluted PPP1R11, PP1γ2, and Sds22 were incubated with anti-PPP1R11, anti-PP1γ2, anti-Sds22, or preimmune serum immobilized on Protein G-Sepharose 4 beads, as indicated at the top of the figure. The immunoprecipitates were separated by SDS-PAGE and immunoblotted for co-precipitating proteins as indicated below each blot strip (The reason why Sds22 migrates as a doublet is not known). *B*. C4 and C5 fractions of testis proteins purified by Superose 6 column chromatography were also separated by native PAGE followed by western blot analysis. Triplicated blot strips probed with either anti-PPP1R11, anti-PP1γ2, or anti-Sds22 antibodies, as indicated, demonstrate that PPP1R11, PP1γ2, and Sds22 co-migrate.

To expand this assessment, we immunoprecipitated these fractions with anti-PPP1R11, anti-PP1γ2, and anti-Sds22 antibodies, and evaluated the immunoprecipitates by SDS-PAGE/western blotting as in Figure 2B. In all cases, antibodies raised against each of the three proteins were able to reciprocally precipitate the other two proteins (Fig. 4A). The fractions containing the co-eluted proteins from the final sizing column were further analyzed by native PAGE/western blotting. Replicate blot strips were separately probed with anti-PPP1R11, anti-PP1γ2, or anti-Sds22 antibodies (Fig. 4B). The results showed that all three proteins co-migrated, thus, offering further evidence that PPP1R11, Sds22, and PP1γ2 are bound to one another in a single complex in testis.

### Estimation of the molecular weight of the complex suggests that it consists of more than three subunits

In a recent study with somatic cells in culture, PP1, PPP1R11, and Sds22 were shown to exist as a trimeric complex in which PP1 was catalytically inactive [29]. To test whether the putative testis complex containing PP1γ2, PPP1R11, and Sds22 was a simple trimer or existed as a larger multimer, we estimated the molecular weight of the chromatographically purified testicular fractions of interest by further size exclusion chromatography through Superdex 200. A calibration curve (data not shown) suggested that the apparent total molecular weight of proteins eluting in these fractions was ∼175 kDa. Based on their primary sequences, the calculated molecular weight of a trimeric complex of just one copy each of PP1γ2, Sds22, and PPP1R11 is ∼96 kDa. This suggests that a complex containing one copy each of the three proteins either migrates anomalously through the sizing column or exists as part of a larger multimeric complex.

To determine if there were components other than PP1γ2, Sds22, and PPP1R11 in the (presumed) larger complex, non-denaturing gel electrophoresis of the Superdex 200 column fraction containing PPP1R11, PP1γ2, and Sds22 was performed as in Figure 4B, but this time in quadruplicate, rather than in triplicate on the same gel (data not shown). Three lanes of the gel were transferred to a PVDF membrane and each was evaluated on a western blot probed with anti-PPP1R11, anti-Sds22, or anti-PP1γ2 antibodies, while the fourth lane of the gel was stained with Coomassie blue. The band in the Coomassie blue-stained portion of the gel corresponding to the common immunoreactive band in the western blots was excised from the gel and microsequenced following in-gel proteolysis. Interestingly, around 15 proteins were identified. Of these, actin was well represented, along with all PP1 isoforms, Sds22, and PPP1R11. Together, the elution profile of the complex on the size-exclusion column and the sequencing results following native gel electrophoresis revealed that the complex containing PPP1R11, Sds22, and PP1γ2 is probably larger than a trimer.

**Figure 5:**
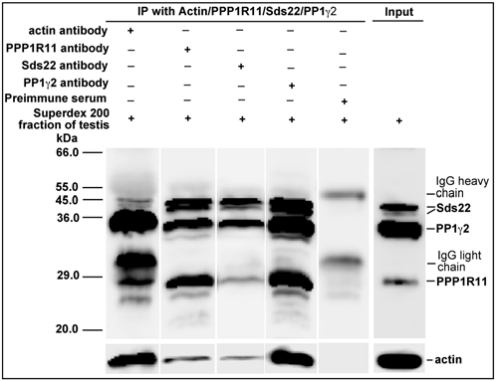
Actin co-precipitates with PP1γ2, Sds22, and PPP1R11 in testis. Fractions from mouse testis protein extracts first purified by DEAE-, MonoQ-, and Superdex 200-column chromatography were immunoprecipitated with anti-actin, anti-PPP1R11, anti-Sds22 and anti-PP1γ2 antibodies. Following SDS-PAGE of the immunoprecipitates, the gels were analyzed by western blotting with antibodies against the proteins indicated to the right of the blots.

Subsequent to microsequencing, chromatographically-purified protein fractions from wild-type mouse testis were immunoprecipitated with anti-actin, anti-PPP1R11, anti-PP1γ2, and anti-Sds22 antibodies. Figure 5 shows that each antibody not only precipitated its cognate antigen, but co-precipitated the other three proteins as well, suggesting that PP1γ2, PPP1R11, Sds22, and actin constitute either the entirety or components of several related multimeric protein complexes in testis.

### PP1γ2 is catalytically inactive in a purified fraction of sperm proteins also containing PPP1R11, Sds22, and actin

**Figure 6:**
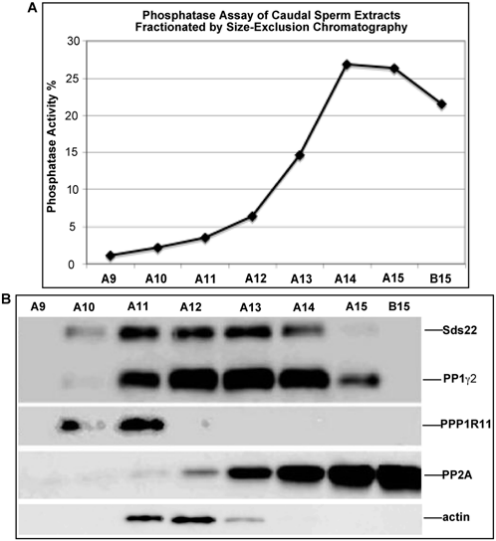
PP1γ2 is inactive in chromatographically-purified fractions containing PP1γ2, Sds22, PPP1R11, and actin. *A*. Protein phosphatase activity in 5-µl aliquots of DEAE-, MonoQ-, and Superose 6-purified fractions from cauda sperm protein extracts. *B*. SDS-PAGE/immunoblot analysis of the corresponding fractions probed with anti-Sds22, anti-PP1γ2, anti-PPP1R11, anti-PP2A, and anti-actin antibodies.

The testis contains all four PP1 isoforms, PPP1R11, and Sds22, as well as abundant PP2A co-eluting with them. Because PP1γ2 is the only PP1 isoform present in cauda epididymal sperm, and PP2A is nearly undetectable in fractions containing PP1γ2, PPP1R11, Sds22, and actin, serine/threonine phosphatase activity was measured in the appropriate chromatographically-purified protein fractions from sperm (Fig. 6A & 6B). These assays revealed that there was little if any serine-threonine phosphatase activity in fractions containing a low level of PP2A and abundant PP1γ2, PPP1R11, Sds22, and actin (Fig. 6B).

### The mutual increase of PPP1R11, PP1γ2, and Sds22 levels in the testis from adolescence to adulthood parallels the progression of haploid male germ cell morphogenesis

**Figure 7:**
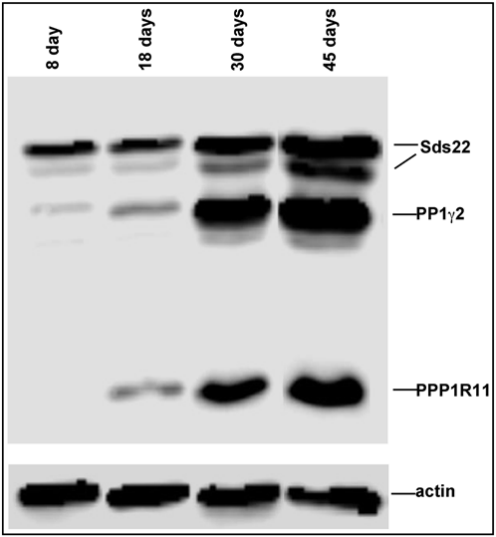
Increasing steady state levels of PPP1R11, PP1γ2 and Sds22 in testis parallel the temporal progression of spermiogenesis. In each experiment, postnatal testis levels of PP1γ2, Sds22, and PPP1R11 were monitored by assessing equal concentrations of protein (25 µg) from extracts prepared from each age group. Proteins were separated by SDS-PAGE, and gels were assayed by western blotting with antibodies against PP1γ2, Sds22, and PPP1R11. Results indicating that postnatal expression of PP1γ2, Sds22, and PPP1R11 increased over the age range in which the initial round of spermiogenesis takes place were visualized by chemiluminescence. A duplicate blot was probed with an anti-actin antibody to demonstrate equal protein loading.

**Figure 8:**
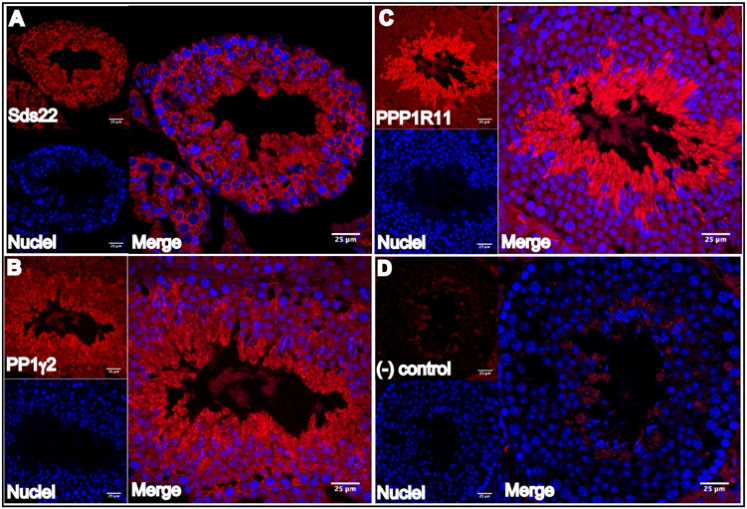
Cellular localization of PP1γ2, PPP1R11 and Sds22 in wild-type mouse testis sections. *A*. Sds22 expression is prominent in the cytoplasm of most germ cells in the testis (from spermatogonia, primary spermatocytes, secondary spermatocytes, and round spermatids to spermatozoa). *B*. PP1γ2 is prominently expressed in the cytoplasms of secondary spermatocytes, spermatids, and mature testicular spermatozoa. *C*. PPP1R11 is prominently expressed in the cytoplasm of haploid germ cells (from round spermatids through spermatozoa). *D*. Immunohistochemistry performed without primary antibody (negative control). Blue fluorescence indicates TO-PRO3 staining of nuclei.

To gain further insight into possible functional relationships between PPP1R11, Sds22, and PP1γ2 in developing male germ cells, the expression and localization patterns of the three proteins in testis were compared. Western blot analysis showed that significant amounts of Sds22 were present in the testis at days 8 and 18 post partum when little PP1γ2 or PPP1R11 was detectable. However, the steady state levels of PP1γ2, PPP1R11, and Sds22, all rose to their highest levels between days 30 and 45 (Fig. 7). This pattern of high level co-expression correlates closely with a time course over which spermatids differentiate into mature testicular spermatozoa. Consistent with this data, immunohistochemical analysis of testis sections confirmed that Sds22 was relatively more abundant in the cytoplasm of spermatocytes than either PP1γ2 or PPP1R11, with PP1γ2 more pronounced than PPP1R11 in these cells (Figs. 8A, 8B, & 8C). PPP1R11 fluorescence intensity in the cytoplasms of round and elongating spermatids and in spermatozoa confirmed that it is primarily a haploid germ cell-restricted protein (Fig. 8C).

### Localization of PPP1R11 in the PP1γ-null testis is essentially wild-type although fluorescence intensity is significantly reduced

**Figure 9:**
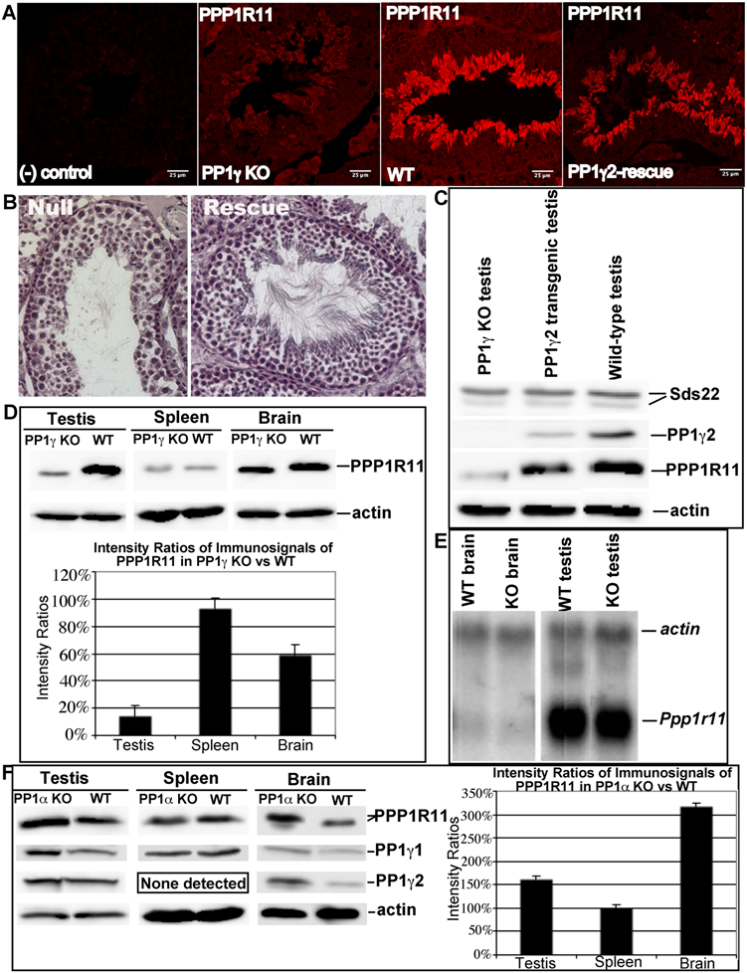
Testicular phenotypes related to the expression of PP1γ2. *A*. Immunofluorescence of PP1γ-null (KO), wild-type (WT), and PP1γ2-rescue testis sections probed with anti-PPP1R11 antibody. Negative (−) control = no primary antibody. *B*. Histological analysis of hematoxalin-stained testis sections from PP1γ-null (Null) and PP1γ2-rescue (Rescue) mice demonstrating an antiapoptotic effect of PP1γ2. *C*. Western blot of testis protein extracts from PP1γ-null (left lane), PP1γ2-rescue (center lane), and wild-type (right lane) animals probed with anti-Sds22, anti-PP1γ2, anti-PPP1R11, and anti-actin antibodies. *D*. Upper panel: PPP1R11 level in PP1γ-null spleen is not significantly diminished compared to its level in wild-type spleen, but is significantly diminished in tissues normally expressing PP1γ2 (testis and brain); Lower panel: histogram of immunosignal ratios of PPP1R11 in PP1γ-null vs wild-type tissues. *E*. Northern blot analysis showing that *Ppp1r11* mRNA levels are comparable in PP1γ-null and wild-type brain and testis, respectively. Actin mRNA levels are used as gel loading control. *F*. Left panel: PP1γ isoform levels are increased and PPP1R11 levels rise dramatically in PP1α-null vs wild-type testis and brain, but PP1γ1 and PPP1R11 levels in PP1α-null spleen are unchanged; Right panel: histogram of immunosignal ratios of PPP1R11 in PP1α-null vs wild-type tissues.

Because PPP1R11 shows haploid germ cell-restricted expression, we performed an immunohistochemical evaluation of its expression pattern in PP1γ-null testis where apoptosis is most pronounced in haploid germ cells [33]. As shown in Figure 9A, the localization of PPP1R11 appeared roughly similar in both PP1γ-null and wild-type testis, though fluorescence intensity was significantly reduced in the former, and the depleted spermatids appeared to be disorganized in PP1γ-null testis, possibly reflecting the apoptotic state of these cells. However, to determine the effect of the absence of PP1γ2 vs. PP1γ1 on the PP1γ-null testicular phenotype, including effects on expression/stability of proteins with which PP1γ2 interacts, we produced PP1γ-null mice expressing transgenic PP1γ2 specifically in the testis (PP1γ2-rescue mice; see Materials and Methods). Immunohistochemical staining of sections from PP1γ2-rescue testis suggested a significant recovery of PPP1R11 expression levels (Fig. 9A). Interestingly, a comparison of histological sections from the testis of PP1γ-null and PP1γ2-rescue mice revealed that PP1γ2 expression in the mutant testis had an obvious anti-apoptotic effect (Fig. 9B).

Western blotting also showed that the steady state level of PPP1R11 was significantly reduced in the PP1γ-null testis, while the levels of Sds22 and actin were essentially unaltered relative to their wild-type levels (Fig. 9C). In contrast, western blot analysis of testis extracts from the PP1γ2-rescue mice showed that the PP1γ2 steady state level was ∼35% of wild-type, and, as expected, the level of PPP1R11 increased, though not quite to wild-type levels, while the levels of Sds22 and actin again appeared unaltered (Fig. 9C).

### The steady state level of PPP1R11 varies directly with the level of PP1γ2 in apoptotic and non-apoptotic tissues

Figure 9C could be interpreted in one of three ways: either (1) the actin/Sds22 levels appear unaltered in the PP1γ-null testis protein extract because increased diploid germ cell/somatic cell proteins relative to haploid germ cell proteins are loaded on the gel due to predominant spermatid apoptosis; (2) the absence of the PP1γ isoforms results in increased translation/stability of Sds22 and/or reduced translation/instability of PPP1R11; or (3) a combination of (1) and (2).

To further explore these options, we focused our attention on the fate of PPP1R11 steady state levels in wild-type and PP1γ-null testis, brain, and spleen (where these tissues in wild-type animals show major, minor, and no PP1γ2 expression, respectively). As demonstrated by western blot analysis, the PPP1R11 level in PP1γ-null testis was reduced to about 14% of wild-type testis PPP1R11 (Fig. 9D). Interestingly, the level of PPP1R11 was also diminished in PP1γ-null brain to ∼59% of wild-type brain PPP1R11, while in PP1γ-null and wild-type spleen, the levels of PPP1R11 were similar (Fig. 9D). It is noteworthy that other than testis, the only tissue expressing the PP1γ2 isoform, albeit at low levels, is brain [34], where no structural, apoptotic, or other neurological phenotype has been reported in PP1γ-null mice. In addition, northern blot analysis showed that *ppp1r11* mRNA is present at comparable levels in wild-type and PP1γ-null testis and brain (Fig. 9E). Together, these results suggest that in haploid male germ cells undergoing morphogenesis, the presence of PP1γ2 could be instrumental in maintaining adequate levels of PPP1R11.

Because the increase in PP1α and PP1β levels in germ cells from PP1γ−null testis [35] did not appear to compensate for the loss of PP1γ isoforms with regard to steady state levels of PPP1R11 in those cells, we studied whether either PP1γ1 or PP1γ2 were up-regulated in PP1α-null testis, brain, or spleen, and if so, would the up-regulation of either or both correlate with any change in PPP1R11 levels. Of note, PP1α-null animals display no overt defect and, thus, appear phenotypically normal (Dr. Angus Nairn, unpublished results). Interestingly, in tissues where both PP1γ isoforms are normally expressed (testis and brain), not only were the levels of both PP1γ isoforms enhanced in PP1α-null mice compared to their wild-type levels, but the steady state level of PPP1R11 was significantly increased over the wild-type level (∼160% and ∼316% respectively in PP1α-null testis and brain; Fig. 9F). However, in PP1α-null spleen, a tissue where PP1γ1 is normally abundant but PP1γ2 is not expressed, PP1γ1 and PPP1R11 levels did not appear to be affected by the loss of PP1α or the absence of PP1γ2 (Fig. 9F). These results further support the idea that the presence or absence of PP1γ2 has a positive effect on the translation efficiency or stability of PPP1R11 in both testis and brain.

### PPP1R11 may be ubiquitinated in PP1γ-null testis

**Figure 10:**
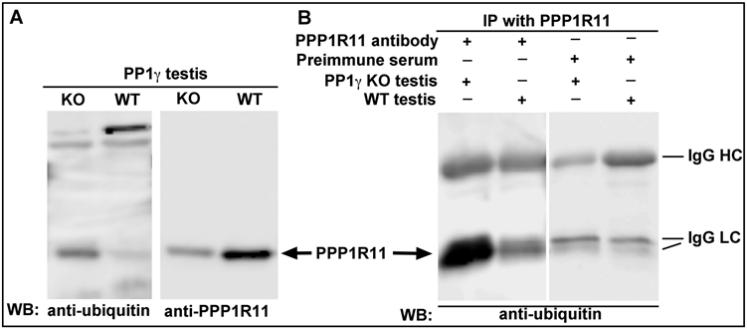
In PP1γ-null testis, PPP1R11 is ubiquitinated or associated with an ubiquitinated protein whose apparent size is equivalent to that of PPP1R11. *A*. Western blot analysis of wild-type and PP1γ-null testis protein extracts probed with either anti-mono- and poly-ubiquitinylated conjugates or anti-PPP1R11 antibodies, as indicated below the blots. *B*. Western blot of anti-PPP1R11 or preimmune serum immunoprecipitates from testis extracts probed with a monoclonal antibody that recognizes mono- and poly-ubiquitinylated conjugates.

PPP1R11 was recently shown to enhance apoptosis via its Caspase3-mediated cleavage and subsequent degradation in actinomyosin D-treated HL60 cells [9]. We have shown that the expression of PP1γ2 in PP1γ-null testis has an anti-apoptotic effect, and appears to enhance the steady state level of PPP1R11 in both apoptotic and non-apoptotic tissues. Because Caspase-mediated apoptosis is known to play an important role in eliminating defective germ cells in the post-natal developing wild-type testis, and proteins cleaved by this process are polyubiquitinated and degraded via the ubiquitin-proteasome pathway [36], we determined the ubiquitinated state of PPP1R11 in wild-type vs. PP1γ-null testis. Western blot analysis with an anti-ubiquitin monoclonal antibody suggested that a protein, with an apparent molecular weight identical to that of PPP1R11 is considerably more ubiquitinated in PP1γ-null testis extracts than in extracts from wild-type testis (Fig. 10A). Additional western blot studies of anti-PPP1R11-immunoprecipitated testis extracts with the same anti-ubiquitin monoclonal antibody demonstrated that either this ubiquitinated protein is a PPP1R11-associated protein in testis or is PPP1R11 (or an N-terminal fragment thereof) itself (Fig. 10B).

## Discussion

Previous studies established that aberrant male germ cell development and testicular apoptosis, particularly during spermiogenesis, were two dominant features of the male sterility phenotype in PP1γ-null mice [33], [35]. Because the latter study showed that PP1γ1 localizes primarily in Leydig (somatic) cells in the testis, we have speculated that the absence of PP1γ2 from developing spermatocytes and spermatids of PP1γ-null mice is the predominant feature underlying the development of structurally abnormal male germ cell characters leading to their apoptotic demise in these mice. Therefore, the present study was initially undertaken to test the assumption that PP1γ2 plays a singular, but as yet unknown role among PP1 isoforms in the development of mammalian male germ cells (particularly spermatids), and to begin to elucidate the biochemical basis of its uniqueness.

Our first goal was to identify potential wild-type testicular interaction(s) of PP1γ2 with its regulators, PPP1R11 (inhibitor 3; I3; previously known as TCTEX5), a highly conserved and potent PP1 inhibitor genetically linked to the male sterility phenotypes of impaired sperm tail development and poor sperm motility in *t* complex mice [37]–[41]; and with PPP1R7 (Sds22), an evolutionarily ancient PP1 regulator bound to PP1γ2 in mammalian caudal epididymal sperm [14]. These prospective relationships were of particular interest because in yeast and in cultured mammalian somatic cells, PP1 isoforms were known to form trimeric complexes with PPP1R11 and Sds22 in which the PP1 isoforms were held in a catalytically inactive state [29], [30].

Our results thus far have indicated that in wild-type testis, PP1γ2, PPP1R11, and Sds22 are present at their highest steady state levels in developing spermatids, and that these three proteins plus actin comprise a tetrameric (or parts of a larger) protein complex. While we could not determine the state of PP1γ2 activity in this testicular complex, we have demonstrated that PP1γ2 is rendered catalytically inactive in its cauda epididymal spermatozoon equivalent. We assume that PP1γ2 inactivity is not an artifact of the protein purification process for the following reasons: (1) our results are in agreement with those of the aforementioned studies showing that PP1-PPP1R11-Sds22 trimers in yeast or somatic cell culture are catalytically inactive [29]; and (2) we used identical techniques here to those employed previously to purify a catalytically active complex of PP1γ2 bound to 14-3-3ζ in sperm [42].

Because the role of a catalytically inactive PP1 complex could be relevant to the preservation of one or more of its component parts for future function in sperm during fertilization, our second goal was to determine the fate of PPP1R11 and Sds22 in PP1γ-null testis and brain. Repeated analyses have established that the steady state protein (but not mRNA) level of PPP1R11 is dramatically diminished in PP1γ-null testis, whereas the levels of Sds22 and actin remain relatively equivalent to their levels in wild-type testis. A simple explanation for this result is that lower PPP1R11 levels reflect the apoptotic loss of spermatids in the PP1γ-null testis, while Sds22 and actin, which are also expressed in earlier germ cells, are upregulated in those cells (or in somatic cells in the case of actin) in the PP1γ-null testis. A test of this explanation, an examination of PPP1R11 and Sds22 levels in PP1γ-null non-apoptotic brain, demonstrated that cell loss in the apoptotic PP1γ-null testis might not completely account for the diminished level of PPP1R11, as significantly diminished PPP1R11 levels and unperturbed Sds22 levels were noted in the non-apoptotic PP1γ-null brain. These results demonstrated a correlation between diminished PPP1R11 levels and the absence of the PP1γ isoforms irrespective of the apoptotic state of cells, a relationship whose probability has been raised by the finding that a significant increase in PPP1R11 levels in the PP1α–null testis and brain correlates with the increased expression of “compensatory” PP1γ isoforms. In addition, in the PP1γ-null and the PP1α–null spleen, a tissue in which PP1γ1 is normally highly expressed, but PP1γ2 is not expressed, PPP1R11 and Sds22 levels are neither diminished nor enhanced, but instead remain essentially wild-type. These results indicate that a primary role for PP1γ2 in cells in which it is expressed could be to enhance the translation efficiency and/or stability of its own inhibitor, PPP1R11. Whether catalysis by an active pool of PP1γ2 or the structure of the inactive PP1γ2-PPP1R11-Sds22-actin complex described here contributes either directly or indirectly to maintenance of a stable steady state level of PPP1R11 in the testis is not known at this time.

We have also demonstrated that the expression of transgenic PP1γ2 can significantly reduce or overcome programmed cell death in the PP1γ-null testis. While admittedly not yet directly tested, it is tempting to speculate that this finding regarding PP1γ2 is either directly or indirectly related to the correlation between its expression and enhanced steady state levels of PPP1R11 in all tissues so far tested. A recent study by Huang and Lee (2008) demonstrated that Caspase-mediated apoptosis in actinomyosin D-treated HL60 cells is controlled at the level of the ability of activated Caspase 3 to recognize PPP1R11 and cleave it at a site located between an upstream PP1 binding site and a downstream PP1 inhibitory site. When this cleavage site on PPP1R11 is altered so that Caspase 3 can no longer sever it, apoptosis in these cells is significantly reduced. Earlier studies have also shown that the cleaved products of Caspase 3-mediated male germ cell apoptosis in immature testis undergoing the primary wave of spermatogenesis are degraded by the ubiquitin-proteasome pathway [36]. With this in mind, we have demonstrated that either PPP1R11 is ubiquitinated and migrates anomalously on SDS gels (the addition of one or more 8.5-kDa ubiquitin polypeptides to PPP1R11 might be masked, since non-ubiquitinated PPP1R11 normally migrates at nearly twice its calculated molecular weight on SDS gels [9]), a ubiquitinated N-terminal fragment of PPP1R11 is present in the PP1γ-null testis (anti-PPP1R11 antibodies used here were raised against PPP1R11 N-terminal peptides), or another ubiquitinated protein of apparent identical size associates with PPP1R11 in PP1γ-null testis.

It remains puzzling why the other PP1 isoforms, PP1α and PP1β, are unable to enhance PPP1R11 levels, even though they are fully capable of interacting with it (and with Sds22 and actin) (data not shown), and appear to increase in amount in PP1γ-null germ cells [35]. One possibility is that the unique and highly conserved C-terminus of the mammalian-specific, testis/sperm-restricted PP1γ2 isoform has evolved to provide this function, whereas the C-termini of the other more evolutionarily ancient isoforms are not designed to do so. This idea is in keeping with the fact that mammalian sperm, in which PP1γ2 exists as the only PP1 isoform, have evolved many novel structural and functional attributes that seemingly would require the evolution of original regulatory mechanisms. We are currently investigating this possibility.

In summary, our studies document, for the first time, the ability of PP1γ2 to form a catalytically-inactive complex with PPP1R11, Sds22, and actin in testicular germ cells and sperm. Furthermore we also show that there exists a reciprocal relationship between the level of PP1γ2, a testis- and sperm-restricted PP1 isoform present only in mammals, and the steady state level of PPPR11.

## Materials and Methods

### Ethics Statement

All procedures involving animals used in this study were approved by the National Institute of Environmental Health Sciences Animal Care and Use Committee and the Kent State University Animal Care and Use Committee.

### Mice Used In This Study

Founder PP1γ-null mice were a kind gift of Dr. Susan Varmuza (University of Toronto, Canada). To produce PP1γ-null mice expressing transgenic PP1γ2 in testis, a human *Pgk2* (phosphoglycerate kinase 2) promoter fragment was used [provided by Dr. John McCarrey (University of Texas, San Antonio, USA)]. This promoter fragment was subcloned into pBluescript SK+ between the restriction sites Sac I and Xba I. Rat testis PP1γ2 cDNA was subcloned downstream of the *Pgk2* promoter fragment between the BamHI and XhoI sites. An SV40 poly A signal was produced by PCR amplification of a pcDNA4.0 plasmid (forward and reverse oligonucleotide PCR primers were: 5′-CTCCTCGAGTCTCATGCTGGAGTTCT-3′ and 5′-CTCGGTACCACCATGATTACGCCAAG-3′, respectively), and the resultant fragment was subcloned downstream of the PP1γ2 cDNA between the XhoI and KpnI sites. The entire fragment containing the *Pgk2* promoter, PP1γ2 cDNA, and SV40 poly A signal was then excised from the pBluescript SK+ vector with BamH1 and KpnI and used for pronuclear injection to produce transgenic mice at the Case Western Reserve University Transgenic Animal Facility (Cleveland, Ohio, USA). PP1γ2 transgenic positive mice in a PP1γ −/− background were obtained by first crossing transgenic founder mice with PP1γ −/− CD1 female mice or PP1γ +/− male mice, then intercrossing PP1γ2 transgenic/PP1γ+/− progeny. The presence of PP1γ2 transgene in a PP1γ-null background and subsequent expression of PP1γ2 were verified by a combination of PCR, RT-PCR, and western blotting. A manuscript describing the complete phenotype of these mice is currently in preparation. Testis, brain, and spleen were also obtained from PP1α-null mice. A manuscript describing the generation of these mice is also in preparation.

### Protein Extract Preparation

Testis, brain, spleen, lung, liver, and heart tissues were homogenized in Buffer B (10 mM Tris-HCl, pH 7.0, 1 mM EDTA, 1 mM EGTA, 10 mM benzamidine-HCl, 1 mM PMSF, 0.1 mM N-tosyl-L-phenylalanine chloromethyl ketone [TPCK], 0.1% [V/V] β-mecaptoethanol). The homogenates were centrifuged at 16,000×*g* to remove insoluble material.

### Western Blot Analysis

Protein extracts boiled in Laemmli sample buffer and separated by 12% SDS-PAGE were electrophoretically transferred to Immobilon-P PVDF membranes (Millipore Corp., Billerica, MA, USA). After blocking non-specific binding sites with 5% nonfat dry milk in Tris-buffered saline (TBS: 25 mM Tris-HCl, pH 7.4, 150 mM NaCl), blots were incubated with primary antibody overnight at 4°C. All primary antibodies were commercially prepared. Anti-PP1γ2 (Zymed Laboratories, San Francisco, CA, USA) was raised against a peptide corresponding to the unique, highly conserved carboxyl terminus of PP1γ2 (amino acid residues 315 to 337, VGSGLNPSIQKASNYRNNTVLYE), 1∶5,000 dilution. The rabbit polyclonal anti-PPP1R11 (Affinity BioReagents, Golden, CO, USA) was raised against two synthetic peptides, the first containing the 1^st^ to 16^th^ amino acid residues (MAETGAGISETVTETT), and the second containing the 27^th^ to 41^st^ amino acid residues (EPENQSLTMKLRKRK), 1∶2,000 dilution. Anti-Sds22 (Affinity Bio Reagents, Golden, CO, USA) was raised against two synthetic peptides, the first containing the 70^th^ to 84^th^ amino acid residues (ETINLDRDAEDVDLC) and the second containing the 347^th^ to 360^th^ amino acid residues (LPSVRQIDATYVRF), 1∶2,000 dilution. Antibodies were affinity purified with the synthetic peptides conjugated to a sulfo-link column (Pierce, Rockford, IL, USA). Anti-mono- and polyubiquitinylated conjugates, monoclonal (clone FK2), was purchased from BIOMOL International, L.P. (Plymouth Meeting, PA, USA), 1∶1,000 dilution. After washing, blots were incubated with anti-rabbit/mouse secondary antibody (1∶2000 dilution) conjugated to horseradish peroxidase (GE Healthcare, Piscataway, NJ, USA) for 1 h at room temperature. Blots were washed with TTBS twice for 15 min each and four times for 5 min each, and then developed with a homemade ECL chemiluminescence kit.

### Expression of Recombinant PPP1R11 in the pGEX vector

We inserted the full-length mouse *PPP1R11* cDNA into the pGEX vector, 4T-2 (GE Healthcare, Piscataway, NJ, USA) and expressed it as a glutathione S-transferase (GST) fusion protein in Escherichia coli. The recombinant protein was subsequently purified on a Glutathione Sepharose 4B affinity column.

### GST Pull-Down Assay

Glutathione Sepharose 4B beads (GE Healthcare, Piscataway, NJ, USA) bound to GST-PPP1R11 or GST alone (as a negative control) were incubated with mouse testis extracts with rocking for 2 h at 4°C. After washing, the proteins were eluted with 20 mM reduced glutathione, 50 mM Tris, pH 8.0. Western blot analysis was performed on the extracts used for pull-down assay and the eluted fractions to identify bound proteins.

### Immunoprecipitation

Crude lysates or purified FPLC fractions of mouse testis were incubated for 1 h at 4°C with ∼5 µg of anti-PP1γ2, anti-PPP1R11, or anti-Sds22 antibody or diluted rabbit pre-immune serum as a negative control. Protein G-Sepharose 4 Fast Flow beads (GE Healthcare, Piscataway, NJ, USA) were washed once with distilled water and twice with TTBS. Each extract/antibody solution was incubated with the beads by rocking for 1 h at 4°C. After incubation, the beads were washed once with TTBS and five times with Buffer B. After washing, the beads were resuspended in 2×SDS reducing sample buffer (6%SDS, 25 mM Tris-HCl pH 6.5, 50 mM DTT, 10% glycerol and Bromphenol blue), boiled for 10 min and centrifuged at 10,000×*g* for 10 min. Supernatants were separated by SDS-PAGE, followed by western blot analysis.

### Column Chromatography

All column procedures were conducted at room temperature following protocols from the manufacturer (GE Healthcare, Piscataway, NJ, USA) on an AKTA FPLC chromatographic system (GE Healthcare, Piscataway, NJ, USA). Mouse testis extracts were first passed through two anion-exchange columns pre-equilibrated with 20 mM Tris-HCl, pH 8.0: a HiTrap DEAE FF (1 ml) column and a Mono Q 5/50 GL (1 ml) column, with proteins eluting in a linear gradient of 0–1 M NaCl in 20 mM Tris-HCl, pH 8.0. Pooled gradient fractions containing the co-eluted proteins, PP1γ2, Sds22 and PPP1R11, from the MonoQ column were passed through the MonoQ column again and proteins were eluted in a linear gradient of 250–550 mM NaCl in 20 mM Tris-HCl, pH 8.0. Subsequentially, the fractions containing the co-eluted proteins were combined and passed through a HiTrap Blue HP column pre-equilibrated with 50 mM NaH_2_PO_4_, pH 7.0. Proteins were eluted in a linear gradient of 0–1.5 M NaCl in 50 mM NaH_2_PO_4_, pH 7.0. The final step of purification involved passing the co-eluting fractions through a size-exclusion column, a Superose 6 10/300 GL column pre-equilibrated with 20 mM Tris-HCl, pH 8.0, 1 mM EDTA and/or a Superdex 200 10/300 GL column pre-equilibrated with 20 mM sodium phosphate, 150 mM NaCl, pH 7.0. The same approach was employed to isolate PPP1R11/PP1/Sds22-containing fractions from mouse brain and PP1γ-null mouse testis. Purified fractions were analyzed either by western blotting with anti-PPP1R11, anti-PP1γ2 and anti-Sds22, by immunoprecipitation, or concentrated with Centricon-10 filters (Millipore Corp., Bedford, MA, USA) for native PAGE analysis. A molecular weight calibration curve for Superdex 200 was generated with the following standards: Catalase (240-kDa), Phosphorylase b (194-kDa), Aldolase (158-kDa), Albumin (68-kDa) and Ovalbumin (43-kDa).

### Native PAGE

Column fractions were mixed with 5× sample buffer (31% [V/V] 1 M Tris-HCl pH 6.8, 5% [V/V] 1% [W/V] bromophenol blue solution, 50% [V/V] glycerol) and separated by electrophoresis through a 10% Tris-HCl Ready Gel (Bio-Rad Laboratories, Hercules, CA, USA) at ∼20–25 mA for about three hours followed by western blot analysis with anti-PPP1R11, anti-Sds22, anti-PP1γ2, or anti-E9 antibodies [E9 antibody: mouse monoclonal anti-PP1c (PP1 catalytic subunit) antibody recognizing all PP1c isoforms from Santa Cruz Biotechnology, Inc. (Santa Cruz, CA)].

### Phosphatase Assay

Phosphatase activity was assayed using a ^32^P-labeled *glycogen phosphorylase a* substrate, as previously reported [2]. Briefly, purified fractions of caudal sperm extracts produced by anion exchange and size-exclusion chromatography were preincubated in a buffer containing 1 mM Mn^2+^ in a total volume of 30 µl at 30°C for 5 min, followed by addition of labeled *phosphorylase a*. At the end of a 10-minute incubation, the reaction was terminated with 95 µl of cold 20% trichloroacetic acid (TCA), after which the reaction tubes were centrifuged for 5 min at 12,000×*g* at 4°C. ^32^PO_4_ counts released from *phosphorylase a* into the supernatants were quantitated. One unit of enzyme activity is defined as the amount of enzyme that catalyzes the release of 1 nmol of ^32^PO_4_ /min.

### Immnohistochemistry of Mouse Testis

Testes were fixed by immersion in 4% paraformaldehyde in PBS at 4°C for 40 h. The fixed testes were transferred to 75% Ethanol and dehydrated, permeabilized, and embedded in paraffin using a Shandon Tissue Processor (Thermo Electron Corp., Waltham, MA, USA). Multiple 8 µm-thick sections of the whole testis were attached to poly-L-lysine-coated slides, deparaffinized, and rehydrated using a standard procedure. Antigen retrieval was performed using 1×Antigen Retrieval Citra Solution (BioGenex, San Ramon, CA, USA). Sections immersed in Citra solution were microwaved for three separate 3-min periods on high setting, with a cooling period of 1 min between each heating cycle. Slides were incubated for 1 h at room temperature in a blocking solution containing 10% normal goat serum in PBS, and then incubated with primary antibodies for 2 h at room temperature or overnight at 4°C. After this, slides were washed three times with PBS, and incubated with corresponding secondary antibody (1∶250) conjugated to indocarbocyanine (Cy3; Jackson Laboratories, West, Grove, PA) for 1 h at room temperature. The slides were washed five times with PBS, mounted with Vectashield (Vector Laboratories, Burlingame, CA, USA) mounting media, and examined using a Fluo View 500 Confocal Fluorescence Microscope (Olympus, Melville, NY, USA). Control slides were processed in the same manner except that the primary antibody incubation was omitted. Cell nuclei were stained with TO-PRO®-3 iodide (Invitrogen, Carlsbad, California) (1∶500) for 20 mins.

### Northern Blot

A 20 µl mixture containing 20 µg of total RNA, 2 µl 10×MOPS buffer, 4 µl HCHO (37% solution, Amresco, OH, USA), 10 µl deionized formamide (Amresco, OH, USA) and 1 µl Ethidium Bromide (200 µg/ml), was heated at 85°C for 10 min, and chilled on ice for another 10 min. A 2 µl aliquot of 10× gel loading buffer (50% Glycerol, 10 mM EDTA pH 8.0, 0.25% w/v bromophenol blue, 0.25% w/v xylene cyanol FF) was added to each sample, and samples were vortexed and briefly centrifuged. Samples were loaded onto 1% agarose/MOPS gel (SeaKem GTG Agarose, Cambrex, NJ, USA), and electrophoresed at 70 V. The gel was denatured in a solution of 0.05 M NaOH and 1.5 M NaCl for 30 min, neutralized in a solution of 0.5 M Tris-HCl, pH 7.4, 1.5 M NaCl, and then equilibrated in 20×SSC. The gel was transferred to Hybond-XL nylon membrane (GE Healthcare, NJ, USA) in 10×SSC for 16 hours. The membrane containing RNA was then baked at 85°C for 2 hours, after which it was prehybridized in 8 ml modified Church buffer (1 mM EDTA, pH 8.0, 0.5 M NaHPO_4_, pH 7.2, and 5%SDS) for one hour at 65°C in a water bath. Fresh Church buffer containing radiolabeled probe (actin cDNA or PPP1R11 cDNA labeled with P^32-^dCTP [MP Biomedicals, OH, USA]) was used for hybridization overnight at 65°C in a water bath. The membrane was then washed in 1×SSC/0.1%SDS three times for 2 min each at room temperature, followed by washing in 0.1×SSC/0.1%SDS twice for 5 min in a 65°C water bath. After washing, the membrane was dried at room temperature, covered with plastic wrap, exposed to film in a cassette with an intensifying screen overnight, and was then developed in a Typhoon™ automated film developer (GE Healthcare, NJ, USA).

### Hematoxylin Staining of Testis

Testes were fixed overnight by immersion in 4% formaldehyde in PBS and then transferred to 70% ethanol and stored at 4°C until processed further. The fixed testis was embedded in paraffin using a Shandon Tissue Processor (Thermo Electron Corp., Waltham, MA, USA). Multiple 8 µm sections were attached to poly L-lysine coated slides and dried overnight at room temperature. The tissue sections were deparaffinized in Citrisolv (Fisher Scientific, Pittsburgh, PA, USA), rehydrated through a graded series of decreasing ethanol percentages in distilled water for 5 minutes. 500 µL of hematoxylin stain (Sigma-Aldrich, St. Louis, MO, USA) was added to each section for 2 minutes, and slides were rinsed in tap water for 2 minutes. The water was replaced once during the rinse. Slides were then dipped 10 times in fresh acid rinse solution (1 ml glacial acetic acid in 49 ml distilled water) and placed in 0.1% NaHCO_3_ for one minute, followed by dipping 10 times in fresh distilled water. Sections were dehydrated through a graded series of increasing ethanol percentages then dipped in Citrisolv for one minute. Excess Citrisolv was wicked, and sections were mounted for histology in a drop of DPX, followed by observation by light microscopy.
